# Y-Site Compatibility Studies of Ketoprofen with Parenteral Nutrition Admixtures for Central and Peripheral Administration

**DOI:** 10.3390/pharmaceutics14122570

**Published:** 2022-11-23

**Authors:** Katarzyna Dettlaff, Aleksandra Gostyńska, Natalia Ziółkowska, Maciej Stawny

**Affiliations:** Chair and Department of Pharmaceutical Chemistry, Poznan University of Medical Sciences, Grunwaldzka 6, 60 780 Poznań, Poland

**Keywords:** ketoprofen, parenteral nutrition, drug compatibility, Y-site, size of lipid droplets, intravenous administration

## Abstract

Ketoprofen (KTF) is often used in hospital wards, especially in its intravenous form. According to the literature review, the compatibility of ketoprofen with parenteral nutrition (PN) admixtures has not yet been investigated. For this reason, we aimed to provide data contributing to physical compatibility to ensure the safe co-administration of these medications. In this study, we examined the compatibility of KTF with eight selected commercial PN admixtures intended for central (Lipoflex Special, Omegaflex Special, Kabiven, SmofKabiven) and peripheral (Lipoflex peri, Omegaflex peri, Kabiven Peripheral, Olimel Peri N4E) administration. The KTF solution for infusion was combined in three different volume ratios with studied PN admixtures reflecting the conditions in clinical practice. The evaluation of undesirable physical destabilization of oil-in-water system or precipitate formation involved the visual inspection and the determination of mean droplet diameter, zeta potential, pH, and turbidity changes. The results of compatibility of KTF with eight commercial PN admixtures showed that three of them: Kabiven, SmofKabiven, and Kabiven Peripheral, are incompatible with KTF and should not be concomitantly administered.

## 1. Introduction

A parenteral nutrition (PN) admixture is a combination of various nutrients administrated to patients via the intravenous route. Patients receiving PN often require concomitant use of other parenteral medications. In the case of limited vascular access, the co-administration of drugs and PN admixtures is almost inevitable. However, the incompatibilities between the components of the PN admixtures and the co-administred drug may occur. Lots of interactions may appear, not only between the individual components but also among the ingredients and the packaging of the drug as well as the excipients contained in both medications [1,2,3,4]. The lack of compatibility between the drug and the PN admixture poses a potential threat to the health and lives of the patients, including capillary embolization and/or lack of pharmacological effect [5,6]. Therefore, co-administration using the Y-site of PN admixtures with another parenteral drug or the addition of drugs to the PN admixtures and their administration from one container must be preceded by analytical tests that confirm their physicochemical compatibility.

Analgesic treatment plays an important role in many therapies. To set standards for pain treatment, the World Health Organization created a general scheme called the analgesic ladder. The most commonly used first-step drugs are diclofenac, ibuprofen, ketoprofen, paracetamol, and metamizole [7,8,9,10]. For surgical patients, there are several methods for the treatment of pain; among those methods, an intravenous infusion is available. There have not been many reports on the stability and compatibility studies of PN admixtures and analgesic drugs. So far, there are only a few reports concerning a simultaneous infusion of analgesic drugs and PN admixtures using a Y-site. The compatibility of paracetamol (10 mg/mL) was most widely investigated with different commercial PN admixtures, including Nutriflex Lipid Special [11], Olimel N5E, Numeta G16E [12], Olimel N5E, Kabiven, and SmofKabiven [13]. In other studies concerning Y-site administration, the compatibility of morphine sulfate (5 mg/mL) with Nutriflex Lipid Special [11], and fentanyl citrate (0.05 and 0.0125 mg/mL), morphine sulfate (1 and 15 mg/mL), and hydromorphone hydrochloride (0.5 mg/mL) with compounded PN admixtures [14] was investigated. Stability studies were conducted by Macias et al. [15]. A 300 mg dose of morphine sulfate and meperidine hydrochloride was mixed with 3000 mL of PN admixture, and the stability of analgesic drugs was determined using the HPLC method [15].

According to the literature review and to the best of our knowledge, the compatibility of KTF with PN admixtures has not yet been investigated. Therefore, this study aimed to determine the physicochemical compatibility of ketoprofen with eight commercial PN admixtures when administered simultaneously using Y-site.

## 2. Materials and Methods

### 2.1. Study Design

Y-site compatibility studies are conducted to establish clinical safety data for the use of simultaneous administration of two parenteral drugs through a single infusion line. In this case, the concomitant administration of KTF and commercial PN admixtures was evaluated. Since physical incompatibility depends on, among other thing, drug concentration [16] and PN admixture composition [17], in this study, the highest concentration of KTF in infusion fluid, recommended by the summary of product characteristics and eight different commercial PN admixtures, was tested [18].

This study can be distinguished by four phases:

Phase I—determination of the physicochemical parameters (pH, osmolality, turbidity, particle size, and zeta potential) of studied drugs, i.e., KTF solution for infusion and eight commercial PN admixtures.

Phase II—calculation of the volume ratios between studied parenteral drugs in order to establish extreme ratios found in the clinical practices.

Phase III—investigation of the physicochemical compatibility between KTF and studied PN admixtures using visual inspection, pH, osmolality, particle size, and zeta potential measurements.

Phase IV—investigation of the interaction between KTF and the water phase of the studied PN admixtures using visual inspection and turbidity measurements.

To consider the PN admixtures to be compatible with the studied drug, the following criteria have to be met. The combination of KTF and PN admixture or KTF and the water phase of the PN admixture must be practically free from visible particles, and no precipitation can be detected upon visual inspection. In the case of the combination of KTF and PN admixture, additionally, no visible sign of lipid emulsion destabilization can be detected. Considering the size of lipid droplets expressed as intensity-weighted (MDD), the studied samples cannot exceed the pharmacopeial limit of 500 nm. This criterion was set by the US Pharmacopeia Method I for the determination of the mean droplet size of injectable lipid emulsions [19]. In the case of pH and osmolality, the change observed during the storage of samples (KTF and PN admixture) has to be below the acceptance criteria of 0.2 units and 5% for pH and osmolality, respectively. In samples combining KTF and the water phase of PN admixtures, the turbidity change between a studied sample and the initial turbidity of each component of this sample cannot exceed the acceptance limit of 0.5 NTU.

### 2.2. Material

Ketonal 50 mg/mL, 2 mL ampoules manufactured by Sandoz GmbH, (Kundl, Austria) was tested.

Eight commercial PN admixtures produced in three-chamber bags were chosen for this study: Lipoflex Special (LS), Omegaflex Special (OS), Lipoflex peri (LP), Omegaflex peri (OP) produced by B. Braun Melsungen AG, Germany; Kabiven (KB), SmofKabiven (SKB), Kabiven Peripheral (KBP) manufactured by Fresenius Kabi AB, Sweden; and Olimel Peri N4E (OLP) purchased from Baxter Polska, Poland. The composition of the PN admixtures is presented in Table S1, and the physicochemical parameters of the KTF solution and PN admixtures are listed in Table 1.

### 2.3. Calculation of the Volume Ratios for Y-Site Compatibility Tests

**Figure 1:**
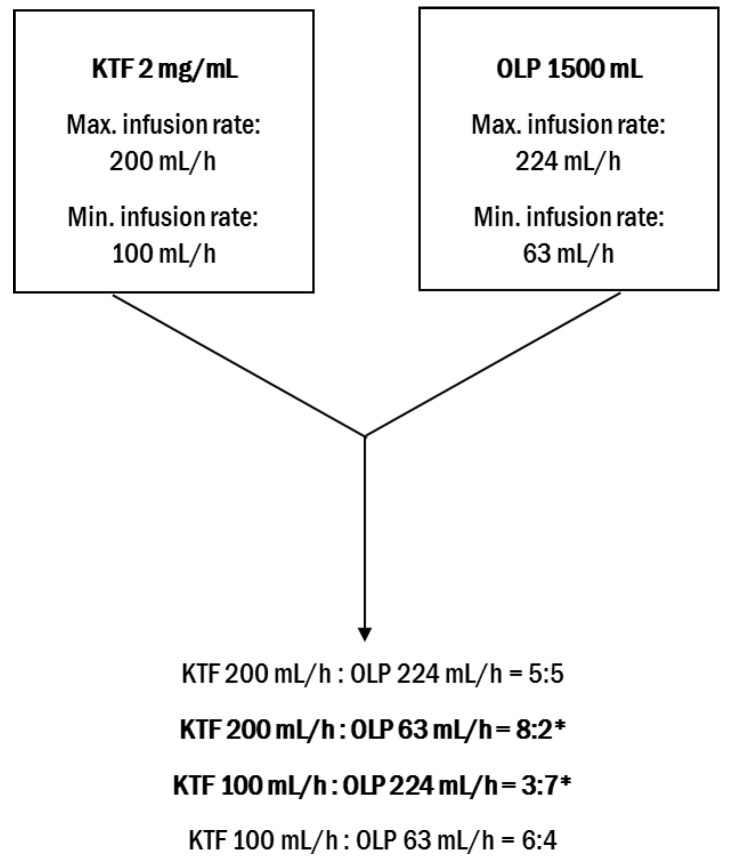
Calculation of the KTF to PN admixtures ratios from the example of Olimel Peri N4E 1500 mL (* extreme KTF-PN ratios).

Based on the summary of product characteristics and the infusion times of the KTF and PN admixture, the KTF:PN admixture volume ratios were calculated. The volume ratios of the KTF and PN admixture in the infusion line were calculated by dividing the drug infusion rate by the PN admixture infusion rate (Figure 1).

On the basis of such a calculation, extreme ratios between KTF and each commercial PN admixture were chosen. The infusion solution of KTF was combined with LS, OS, and SKB in 8:2 and 4:6 volume ratios; in the case of KB, LP, OP, and OLP, ratios 8:2 and 3:7 were investigated, and in the case of a combination of KTF with KBP, ratios 8:2 and 2:8 were investigated. Additionally, an equal volume of drug and PN admixtures were used as it has previously been verified as a standard practice [20].

### 2.4. Sample Preparation

Two ampoules of Ketonal Sandoz were diluted to 100 mL with 0,9% sodium chloride solution (B. Braun Melsungen AG, Melsulgen, Germany) to obtain the concentration of 2 mg/mL, which is the highest drug concentration allowed by the summary of product characteristics to be administered to patients. Each PN admixture was activated according to the manufacturer’s instructions, and then vitamins and trace elements were added as followed. One vial of Viantan (B. Braun Melsungen AG, Melsulgen, Germany) and one ampule of Tracutil (B. Braun Melsungen AG, Melsulgen, Germany) were added to LS, OS, LP, and OP. One vial of Cernevit (Baxter, Warsaw, Poland) and one ampoule of Tracutil were added to OLP (Baxter, Poland). The Soluvit N (Fresenius Kabi AB, Uppsala, Sweden) was dissolved in Vitalipid N Adult (Fresenius Kabi AB, Uppsala, Sweden), and such vitamin emulsion was added to KB, SKB, and KBP together with one ampoule of Addamel N (Fresenius Kabi AB, Uppsala, Sweden). Immediately after the activation of PN admixtures, the samples for the compatibility test were prepared by mixing the appropriate volume of drug and PN admixtures in a 10 mL test tube and stored at a room temperature of 23 ± 1 °C for 4 h to simulate Y-site administration. The compatibility assays, which include a visual examination and determination of pH, osmolality, particle size, and zeta potential, were performed immediately after mixing and after 4 h of storage.

For turbidity assays, lipid-free samples were prepared. For this purpose, two of the three chambers of the PN admixtures bag were activated, resulting in the forming of the water phase of PN admixtures without lipid emulsion. To maintain the concentrations of all components on the same level, the volume of lipid emulsion was replaced with the same volume of water for injection. Such solutions were supplemented with an appropriate pharmaceutical preparation of trace elements and were subject to turbidity assays. Immediately after the preparation of the water phase of PN admixtures, the samples for the turbidity test were prepared by mixing KTF and PN admixtures in selected ratios in a 10 mL test tube. Turbidity measurements were performed immediately after mixing and after 4 h of storage at a temperature of 23 ± 1 °C.

### 2.5. Visual Inspection

Visual inspection was performed according to European Pharmacopoeia requirements described in chapter 2.9.20 [21]. Samples were gently swirled to remove air bubbles and observed for 5 s against a black and white background by two independent observers. Any sign of visible particulates was noted.

### 2.6. Determination of pH, Osmolality, and Turbidity

The pH was determined using a pH-meter (Mettler Toledo Seven Compact pH/ion S220 pH meter, Mettler, Toledo, OH, USA), which was calibrated at two points against standard buffers at pH 4.0 and 7.0. The pH of all samples was measured at the temperature of 23 ± 1 °C. The osmolality was determined by the cryoscopy method using an 800 CL TridentMed osmometer (Trident Med s.c., Warsaw, Poland). pH and osmolality were determined for each sample immediately after preparation and after 4 h of storage. Additionally, the initial values of pH, and osmolality were determined for KTF solution for infusion (Ketonal Sandoz in 0.9% sodium chloride solution, 2 mg/mL) and all studied activated and supplemented PN admixtures.

The turbidity was determined using a Hach TU5200 turbidimeter. Before the measurements, the apparatus was calibrated according to the manufacturer’s instructions against the formazin turbidity standards 20 NTU and 600 NTU and verified using the 10 NTU standard. The turbidity was determined for all tested combinations of KTF and the water phase of PN admixtures immediately after preparation and after 4 h of storage. Additionally, to determine the initial values of turbidity for tested drugs, the turbidity of KTF solution for infusion (Ketonal Sandoz in 0.9% sodium chloride solution, 2 mg/mL) and the water phase of PN admixtures was determined.

### 2.7. Determination of Particle Size and Zeta Potential

The particle size of lipid emulsion and zeta potential of studied samples were measured at the temperature of 25 °C using a Zetasizer Nano ZS (Malvern Instruments, Malvern, U.K.) by dynamic light scattering (DLS) and laser doppler electrophoresis (LDE), respectively. The sample preparation, particle size, and zeta potential determination were performed according to the methodology described in our previous work [22]. The results of droplet diameter measurements were presented as MDD, dF1 (the diameter of the particles present in the highest intensity in the first fraction), and dF2 (the diameter of the particles present in the highest intensity in the second fraction). Particle size and zeta potential determination were performed for all tested samples immediately after preparation and after 4 h of storage as well as for each studied commercial PN admixture just after activation and supplementation with vitamins and trace elements.

### 2.8. Statistical Analysis

All measurements were performed in triplicate, and the results were expressed in terms of the mean values with standard deviation (mean ± SD). The data were analyzed using Statistica 12 software (StatSoft Polska Sp. Z o.o., Cracow, Poland).

## 3. Results

Compatibility studies were performed with eight different commercial PN admixtures: four dedicated to central administration and four designed for peripheral administration. A visual examination of all PN admixtures during the study did not show any signs of lipid emulsion degradation, such as color alteration or phase separation. PN admixtures were combined with KTF diluted in 0.9% sodium chloride. The pH of the KTF solution was 5.57 and did not deviate from the pH of tested PN admixtures (5.54–5.77), with the exception of the OPL, whose pH was higher and was equal to 6.58. The osmolality of KTF was 598 mOsm/kg, whereas the osmolality of the PN admixtures ranged from 800 to 907 mOsm/kg, and from 1160 to 1921 mOsm/kg for central and peripheral PN admixtures, respectively. The MDD was the lowest for the SKB (243.3 nm) and the highest for KB (281.5 nm), and the zeta potential of all PN admixtures ranged from −8.8 mV to −14.5 mV (Table 1). Combining PN admixtures with KTF led to the pH, osmolality, and zeta potential changes that correlated to the proportion of the drug to the PN admixture (Table 2).

**Figure 2:**
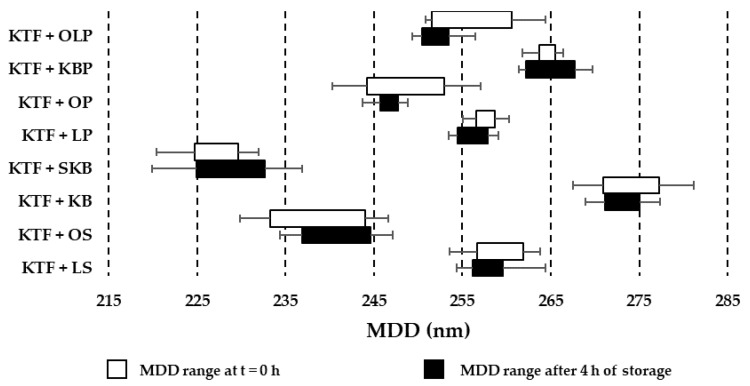
Results of MDD of lipid emulsion ranges for KTF and parenteral nutrition admixtures combinations.

Four hours of storage at the temperature of 23 ± 1 °C in most cases does not significantly affect the tested samples. Only in the case of the KTF and KB combination in an 8:2 ratio does the pH decrease by 0.1 units; in other cases, the pH fluctuations did not exceed this value. The osmolality changed the most in the KTF and SKB combination in the 5:5 ratio, for which the increase in osmolality was 3% of the initial value. The addition of KTF to PN admixtures resulted in MDD changes (Figure 2).

**Figure 3:**
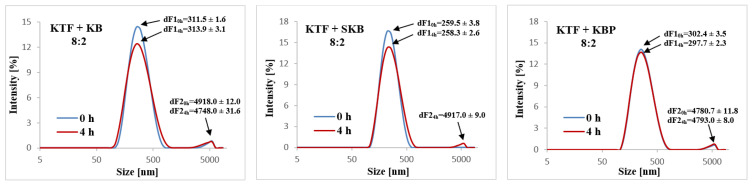
Distribution of the lipid droplets size for KTF and KB, SKB, and KBP combinations where second fraction of particles greater than 4 µm were observed.

The highest value of MDD was recorded for KTF and KB combinations and the lowest for KTF and SKB combinations (Figure 2). Immediately after adding the drug to KB and KBP in the proportion of 8:2, the appearance of the second fraction of particles with a diameter greater than 4 µm was observed (Figure 3). In the case of the KTF and SKB combined in a ratio of 8:2, the second fraction of particles greater than 4 µm appeared after 4 h of storage.

**Figure 4:**
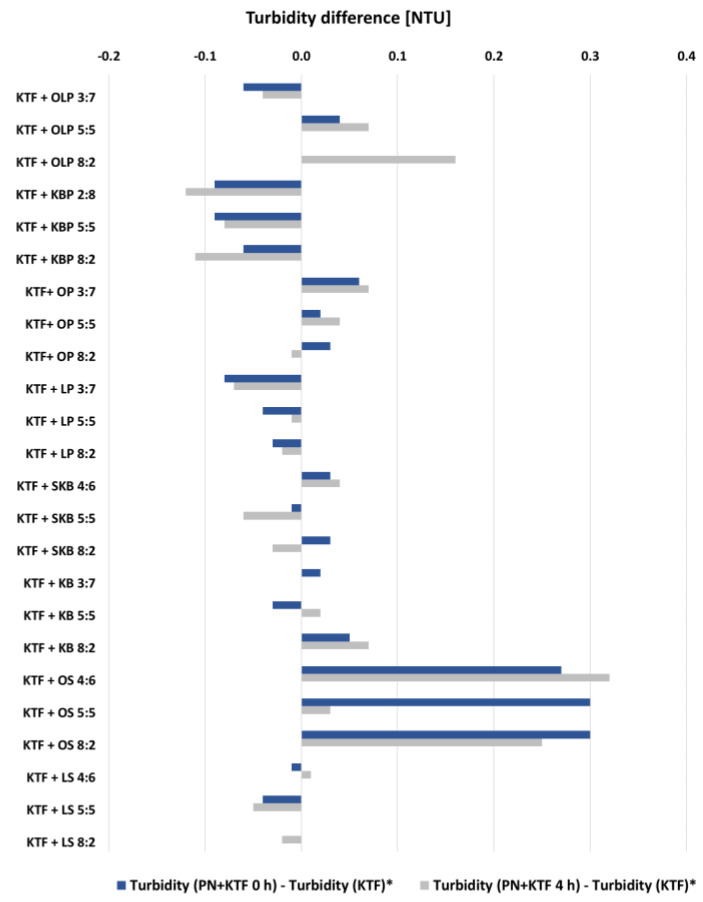
Difference between the turbidity of tested samples at t = 0 h and t = 4 h and the turbidity of KTF. * in the case of KTF + SKB combinations instead of the KTF turbidity the SKB turbidity was subtracted.

To identify the interaction between KTF and the water phase of the PN admixtures, a turbidity evaluation was performed. The turbidity of the KTF solution was 0.26 NTU, and the water phase of PN admixtures was in the range of 0.11 to 0.64 for KPB and SKB, respectively. Combinations of KTF and the water phase of PN admixtures immediately after mixing and after the following 4 h were determined. Afterward, the differences between the turbidity values for all combinations and the upper initial value of either KTF solution or the water phase of PN admixtures were calculated. Only in the case of SKB, the initial turbidity of PN admixture (0.64 NTU) exceeded the turbidity of KTF (0.26 NTU). The results of turbidity differences are presented in Figure 4. The highest difference in turbidity (>0.3 NTU) was observed for the combination of KTF and OS in all tested ratios at the t = 0 h and in ratios 8:2 and 4:6 at the t = 4 h. The increase in turbidity above 0.1 NTU was also observed for the KTF and OLP combination in an 8:2 ratio after 4 h of storage.

## 4. Discussion

**Figure 5:**
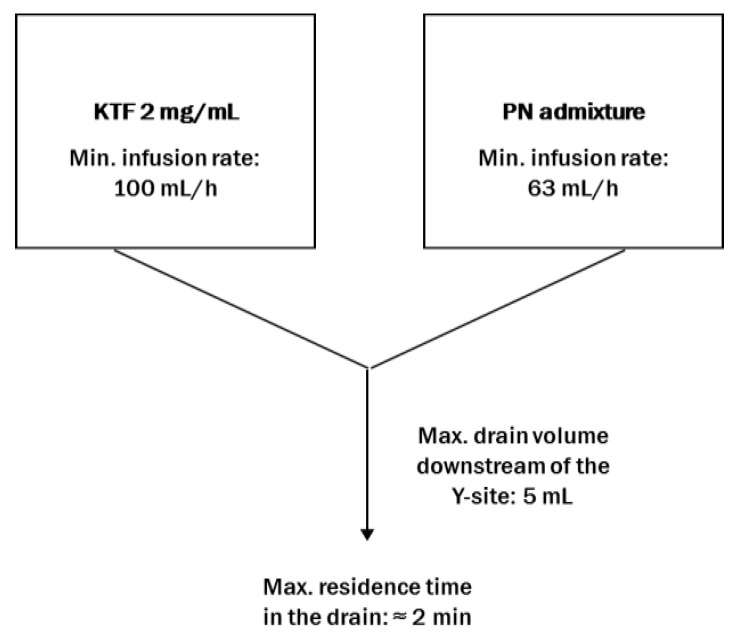
Diagram showing the method of calculating the maximum residence time in drain downstream of the Y-site.

Simultaneous administration of drugs and PN admixtures may be performed using a Y-site connector, which is located in the lower part of the infusion line just before the distal end of the infusion line. In order to calculate the maximum contact time of the drug with the PN admixture, the minimum infusion times of both drugs and the volume of the drain downstream of the Y-site were determined (Figure 5). The maximum contact time of the drug and PN admixture during the simultaneous infusion during Y-site administration is less than 2 min. Considering other authors’ methodology [11,13,23,24] and our own experiences [25,26,27], the chemical instability of KTF was considered low risk due to the short contact time and was therefore not studied in this investigation. To ensure safe administration of combined drugs to patients, a short contact time for the two drugs was administered simultaneously using the Y-site, where physicochemical changes may occur, and the proportion between drugs in the infusion line should be considered since the concentration of each compound may play a crucial role in the compatibility determination. The drug-to-PN admixture ratios can be experimentally determined using infusion pumps connected to the infusion line [28] or can be calculated on the basis of the infusion rate of both medications. The extreme proportion between both drugs and 1:1, which is a standard proportion used by other authors [29], were chosen for the study. The time of examination and storage period set on 4 h were in line with our previous studies [25,26,27] and review of the literature [13].

The commercial PN admixtures selected for the compatibility studies had varying compositions (Table S1). PN admixture manufacturers produce them in several volumes to best suit the different needs of patients. Depending on the composition, PN admixtures may be intended for central (LS, OS, KB, SKB) or peripheral (LP, OP, KBP, OLP) administration and are characterized by different energy densities. Moreover, the commercial PN admixtures differed in the composition of the oil phase of lipid emulsion, which can be a pure soybean oil (KB, KBP) or even a mixture of four different oils (SKB).

Combining drugs with PN admixtures may affect the stability of lipid emulsion, leading to visual changes such as coalescence or phase separation as well as modification of physicochemical parameters such as pH, osmolality, zeta potential, or MDD [6,30].

The literature review allows us to establish acceptance criteria for each parameter. According to Greenhill et al., the changes in pH value during the time above 1 unit should be considered a sign of the chemical reaction occurring in the sample [22]. However, in our previous works, a difference higher than 0.2 units after 4 h of storage was considered a sign of incompatibility [31]. In the interest of safety practice, we have also remained in this work with this restrictive criterion. The pH of all combinations of the KTF and PN admixtures during the 4-h storage did not change by more than 0.1 units, which indicates that the set criterion was met. Osmolality is the measure of solute concentration. The changes in this value over time indicate the chemical reaction that occurs between solutes of PN admixtures and added drugs. Following our previous work, we established the acceptance criterion of changes occurring during storage at ±5% [25,26]. Analyzing the studied samples, it was found that the osmolality of all samples during storage varied within ± 3%, indicating that this criterion was also met.

The zeta potential is a helpful parameter allowing for estimating the stability of the dispersion system. The higher the absolute value of zeta potential, the greater the stability of the emulsion. However, there is no critical value established for this parameter for PN admixtures that eliminate the possibility of its administration to the patient. It is assumed that stable lipid emulsions should have a zeta potential of −30 mV to −50 mV [32]; however, it has been found that the addition of amino acids, glucose, and electrolytes to the system lowers the absolute value of this parameter, i.e., the zeta potential value depends on the composition of the PN admixture [33,34]. Combining PN admixtures with KTF solution for infusion lowered the concentrations of amino acids, glucose, and electrolytes (except for sodium chloride, whose centration increased due to the presence of this salt in KTF solution for infusion). PN admixtures selected for this study, despite having a zeta potential of −8 mV to −15 mV, have a shelf life guaranteed by the manufacturers of at least 24 h at room temperature and up to 7 days when stored in the refrigerator. Therefore, in this study, we only determined changes in the zeta potential during the 4 h after combining the drug with the PN admixture to identify if zeta potential changes result in visual, MDD, or turbidity changes. Despite significant changes in the absolute value of this parameter in some samples, they were not considered incompatible unless there were other changes in physicochemical properties occurred.

One of the most important parameters for the safety of a patient receiving PN admixtures is the particle size of the lipid emulsion. The pharmacopeial criterion set on MDD is 500 nm [19]. None of the tested samples exceeded this criterion immediately after preparation or after 4 h of storage. However, according to McClements’ findings, larger emulsion droplets are more susceptible to destabilization mechanisms of creaming, heterogeneous coalescence, and phase separation [35]. The MDD of the lipid emulsion may slightly change after combining the drug with PN admixture, which is related to the measurement method used. The DLS technique determined the hydrodynamic diameter of the particle, which is slightly larger than the particle diameter itself, and it is influenced by parameters such as diffusion rate, temperature, and viscosity of the sample. Therefore, the change in the environment in which the lipid droplet is suspended, resulting from the addition of KTF solution, may explain slight changes in lipid droplet size in studied samples. Nevertheless, in the case of the KB and KBP, particles of the size above 4 µm (dF_2_) appeared just after the addition of the drug to PN admixtures in the ratio of 8:2. In the case of the SKB in the same drug to PN admixtures ratio, the dF_2_ of about 5 µm was observed, but only after 4 h of storage.

PN admixtures are composed of over 50 active ingredients: amino acids, glucose, fatty acids, electrolytes, trace elements, vitamins, and auxiliary ingredients [6]. Therefore, it is difficult to assess which ingredient interacts with KTF and why after adding a KTF solution in a concentration of 2 mg/mL in 0.9% sodium chloride solution in a ratio 8:2, the second fraction of lipid particles appears in the KB, KBP, and SKB. Analyzing the compositions of PN admixtures, which appear to be incompatible with KTF when administered using the Y-site, it turns out that they do not differ either by the product of Ca^2+^ × P0_4_^3−^ concentrations, the CAN (critical aggregation number) coefficient [36], or the concentration of active substances expressed by theoretical osmolality. Among the PN admixtures selected for the study, KBP has the lowest theoretical osmolality, whereas KB is characterized by the intermediate value of this parameter, i.e., the lowest among PN admixtures for central administration but higher than for mixtures for peripheral administration. SKB, on the other hand, has one of the highest osmolality, but there are PN admixtures that have this higher value (LS and OS). It should be noted that the PN admixtures selected for the tests also differed in terms of the composition of the lipid emulsion. The auxiliary substances used for the production of lipid emulsions were also analyzed. KB and KBP contain purified egg phospholipids (emulsifier), glycerol (co-emulsifier), sodium hydroxide, and glacial acetic acid (for pH adjustment). Additionally, to maintain the substance, SKB contains all-rac-α-tocopherol, and sodium oleate. However, all those auxiliary substances are also present in other studied commercial PN admixtures. Although the same emulsifiers and stabilizers are used to prepare the emulsion, we do not know their concentrations, which are the manufacturers’ classified data; perhaps this is the answer why after adding KTF to 3 out of 8 tested PN admixtures, lipid emulsion particles exceeding 4 µm appear.

In order to explain this phenomenon, additional turbidity assays were performed. For this purpose, KTF was combined in assumed proportions with PN admixtures devoid of lipid emulsion. Following other researchers, we established the acceptance criterion for turbidity change as 0.5 NTU [37]. We calculated the change in turbidity for both the difference between the initial values and the turbidity of the combination at t = 0 and after 4 h of storage. Since the PN admixtures showed varying turbidity, which in one case (SKB) exceeded the value observed for the KTF solution, to calculate the change in turbidity, the higher value of turbidity obtained for the initial preparations was subtracted from the value obtained for the combination of KTF and PN admixtures. In none of the tested samples does the turbidity difference exceed the value of 0.3 NTU. The highest turbidity differences were observed for KTF + OS. In the case of combinations of KTF with KB, KBP, or SKB, the turbidity did not increase by more than 0.1, and in the case of KBP in all tested ratios and time, it even decreased. The lack of sedimentary reactions between the components of the aqueous phase of the PN admixtures and the drug solution was confirmed by turbidimetric measurements. These results clearly indicate that the ingredients of the lipid emulsion and not the aqueous phase of the tested PN admixtures are responsible for the interaction observed between KTF and KB, KBP, and SKB.

## 5. Conclusions

In this study, the compatibility of KTF with eight commercial PN admixtures was examined, showing that three of them: KB, KBP, and SKB are incompatible. Since the turbidity assays showed no sedimentary reactions between the components of the aqueous phase of the PN admixtures and the drug solution, the lipid emulsion components seem responsible for KTF and PN admixtures interaction. Further analysis should be performed to identify the reason for such incompatibilities.

## Figures and Tables

**Table 1:** Infusion rates and physicochemical parameters of tested drug and parenteral nutrition admixtures.

Product name	Min. Infusion Rate [mL/h]	Max. Infusion Rate [mL/h]	pH	Osmolality [mOsm/kg]	Turbidity [NTU]	MDD [nm]	Zeta Potential [mV]
**Drug**							
Ketonal Sandoz in 0.9% sodium chloride solution (2 mg/mL)	100 **	200 **	5.57	598	0.26	n.a.	n.a.
**Parenteral nutrition admixtures for central administration**							
Lipoflex Special 1875 mL	78 *	119 **	5.56	1921	0.20	252.1	−9.2
Omegaflex Special 1875 mL	78 *	119 **	5.56	1906	0.23	258.0	−10.5
Kabiven 1540 mL	64 *	182 **	5.57	1160	0.10	281.5	−8.9
SmofKabiven 1970 mL	82 *	140 **	5.64	1734	0.64	243.5	−8.8
**Parenteral nutrition admixtures for peripheral administration**							
Lipoflex peri 1875 mL	78 *	175 **	5.54	900	0.18	251.0	−12.5
Omegaflex peri 1875 mL	78 *	175 **	5.56	907	0.25	268.1	−14.5
Kabiven Peripheral 1920 mL	80 *	259 **	5.77	800	0.11	265.0	−13.5
Olimel Peri N4E 1500 mL	63 *	224 **	6.58	842	0.19	261.0	−14.2

* calculated by dividing parenteral nutrition admixtures volume by 24 h (the maximum infusion time), ** established on the basis of the summary of product characteristics; MDD—intensity-weighted mean droplet diameter; n.a.—not applicable.

**Table 2:** Physicochemical properties of the combination of KTF and PN admixtures.

Sample	Ratio	pH		Osmolality ± SD [mOsm/kg]		Zeta Potential ± SD [mV]	
		0 h	4 h	0 h	4 h	0 h	4 h
KTF + LS	8:2	5.61	5.59	835 ± 4	839 ± 3	−14.0 ± 1.0	−18.0 ± 0.3
	5:5	5.55	5.56	1207 ± 8	1202 ± 2	−10.5 ± 0.2	−11.1 ± 0.8
	4:6	5.58	5.54	1330 ± 2	1334 ± 2	−8.3 ± 0.3	−9.3 ± 0.5
KTF + OS	8:2	5.63	5.66	851 ± 7	849 ± 1	−18.2 ± 0.6	−18.2 ± 0.6
	5:5	5.61	5.62	1223 ± 4	1211 ± 11	−17.4 ± 0.8	−17.4 ± 0.8
	4:6	5.60	5.61	1348 ± 7	1332 ± 8	−12.8 ± 0.5	−12.8 ± 0.5
KTF + KB	8:2	5.74	5.64	736 ± 6	730 ± 1	−37.3 ± 3.0	−22.1 ± 1.0
	5:5	5.68	5.62	889 ± 2	886 ± 6	−18.5 ± 1.3	−20.1 ± 0.7
	3:7	5.62	5.61	989 ± 9	1000 ± 3	−17.2 ± 0.9	−14.5 ± 0.5
KTF + SKB	8:2	5.70	5.72	837 ± 2	862 ± 4	−21.7 ± 0.4	−21.9 ± 0.5
	5:5	5.68	5.66	1129 ± 7	1163 ± 5	−16.0 ± 0.6	−15.3 ± 0.5
	4:6	5.69	5.68	1237 ± 4	1297 ± 2	−14.4 ± 0.6	−13.6 ± 0.5
KTF + LP	8:2	5.69	5.69	691 ± 5	690 ± 2	−28.7 ± 0.5	−24.4 ± 0.4
	5:5	5.63	5.66	772 ± 2	768 ± 3	−14.8 ± 0.4	−14.2 ± 0.2
	3:7	5.58	5.62	825 ± 3	823 ± 4	−13.0 ± 0.9	−14.1 ± 1.0
KTF+ OP	8:2	5.66	5.68	685 ± 0	685 ± 5	−17.5 ± 0.9	−20.1 ± 0.5
	5:5	5.62	5.65	775 ± 2	772 ± 2	−14.1± 0.5	−14.0 ± 0.5
	3:7	5.59	5.61	827 ± 1	827 ± 4	−13.5 ± 0.9	−13.5 ± 0.3
KTF + KBP	8:2	5.81	5.82	655 ± 4	655 ± 3	−23.8 ± 0.7	−21.0 ± 1.0
	5:5	5.81	5.80	710 ± 3	706 ± 1	−21.4 ± 0.4	−19.2 ± 0.5
	2:8	5.80	5.78	761 ± 6	759 ± 4	−14.5 ± 0.5	−14.9 ± 0.9
KTF + OLP	8:2	6.49	6.49	676 ± 3	676 ± 3	−21.8 ± 1.7	−26.0 ± 1.5
	5:5	6.56	6.54	742 ± 3	745 ± 3	−23.3 ± 0.7	−18.9 ± 0.6
	3:7	6.56	6.55	789 ± 6	787 ± 4	−17.3 ± 0.4	−19.5 ± 0.4

SD standard deviation; SD of pH values of all samples were below 0.02.

## Data Availability

The data is contained in the article and Supplementary Materials.

## Supplementary Materials

The following supporting information can be downloaded at: https://www.mdpi.com/article/10.3390/pharmaceutics14122570/s1, Table S1: Composition and some parameters of studied RTU parenteral nutrition admixtures.
