# Supplementary material for: Y-Site Compatibility Studies of Ketoprofen with Parenteral Nutrition Admixtures for Central and Peripheral Administration
Source: Pharmaceutics. 2022 Nov 23;14(12):2570. doi: 10.3390/pharmaceutics14122570 (PMC9781255; doi:10.3390/pharmaceutics14122570)
Supplement: Supplementary file 1 [file pharmaceutics-14-02570-s001.zip › pharmaceutics-1982167-supplementary.pdf]

Table S1. Composition and some parameters of studied RTU parenteral nutrition admixtures

Ingredient/ parameter	Unit	Central access				Peripheral access			
		**LS**	**OS**	**KB**	**SKB**	**LP**	**OP**	**KBP**	**OLP**
Amino acids	g/L	56	56	33	51	32	32	23.8	25.3
Carbonates		144	144	97	127	64	64	67.5	75
Lipid emulsion		40	40	39	38	40	40	35.4	30
Natrium	mmol/L	53.6	53.6	31.2	41.0	40	40	22.1	21
Potassium		37.6	37.6	23.4	30.0	24	24	16.7	16
Magnesium		4.2	4.2	3.9	5.1	2.4	2.4	2.8	2.2
Calcium		4.2	4.2	1.9	2.5	2.4	2.4	1.4	2.0
Phosphate		16.0	16.0	9.7	13.0	6.0	6.0	7.5	8.5
Energy	kcal/L	1181	1180	877	1100	765	765	708.3	700
Theoretical osmolarity	mOsm/L	1545	1545	1060	1500	840	840	750	760
CAN	mmol/L	631	631	426	560	373	373	308	306
$Ca^{2+} \times P0_4^{3-}$	$mmol^2/L^2$	67	67	18	33	14	14	11	17
Manufacturer	-	B. Braun Melsungen AG, Germany	B. Braun Melsungen AG, Germany	Fresenius Kabi, Bad Homburg, Germany	Fresenius Kabi, Bad Homburg, Germany	B. Braun Melsungen AG, Germany	B. Braun Melsungen AG, Germany	Fresenius Kabi, Bad Homburg, Germany	Baxter, Lessines, Belgium
LOT	-	204328231	191518231	10NH1693	10PA7121	201068231	200528231	10PC9342	20B27N21

CAN — critical aggregation number (CAN = concentration of monovalent cations + 64 × concentration of divalent cations + 729 × concentration of trivalent cations [mmol/L]); $Ca^{2+} \times P0_4^{3-}$ — product of calcium and phosphate concentration [$mmol^2/L^2$]
